# Effectiveness of anti-osteoporotic treatment after successful parathyroidectomy for primary hyperparathyroidism: a randomized, double-blind, placebo-controlled trial

**DOI:** 10.1007/s00423-019-01815-9

**Published:** 2019-08-26

**Authors:** Martin B. Niederle, Ursula Foeger-Samwald, Philipp Riss, Andreas Selberherr, Christian Scheuba, Peter Pietschmann, Bruno Niederle, Katharina Kerschan-Schindl

**Affiliations:** 1 Department of Surgery, Division of General Surgery, Medical University of Vienna, Waehringer Guertel 18-20, 1090 Vienna, Austria; 2 Department of Anesthesia, General Intensive Care and Pain Management, Medical University of Vienna, Waehringer Guertel 18-20, 1090 Vienna, Austria; 3 Department of Pathophysiology and Allergy Research, Center for Pathophysiology, Infectiology and Immunology, Medical University of Vienna, Waehringer Guertel 18-20, 1090 Vienna, Austria; 4 Department of Physical Medicine, Rehabilitation and Occupational Medicine, Medical University of Vienna, Waehringer Guertel 18-20, 1090 Vienna, Austria

**Keywords:** Primary hyperparathyroidism, Parathyroidectomy, Bone mineral density, Osteoporosis, Biomarkers

## Abstract

**Purpose:**

After successful surgery for primary hyperparathyroidism, bone mineral density (BMD) does not improve equally in all patients. As no trial has so far aimed to influence normalization of BMD, it was the goal of this investigation to determine whether pharmacological treatment is effective in improving regain of BMD after successful parathyroidectomy in patients with preoperatively diagnosed osteoporosis or osteopenia and to evaluate when treatment may be indicated.

**Methods:**

In this randomized, placebo-controlled, double-blind trial, 52 patients were treated with strontium ranelate 2 g daily + 1000 mg calcium + 800 IU vitamin D (strontium group; SG) or with 1000 mg calcium + 800 IU vitamin D alone (placebo group; PG) for 1 year. The main outcome measures were BMD (lumbar spine, femoral neck, radius) and bone turnover markers.

**Results:**

The baseline characteristics were similar in both groups. Absolute BMD (1.007 ± 0.197 vs. 0.897 ± 0.137 g/cm^2^; *p* = 0.024) and both relative (9.94 vs. 3.94%; *p* < 0.001) and absolute (0.09 ± 0.06 vs. 0.03 ± 0.04 g/cm^2^; *p* < 0.001) changes in lumbar-spine BMD were significantly higher in the SG than in the PG. Compared to baseline, BMD significantly increased in both groups at the lumbar spine (*p* < 0.001 and *p* = 0.001, respectively) and femoral neck (both *p* < 0.001), whereas radius BMD only changed significantly in the SG. However, the proportion of patients with osteoporosis/osteopenia significantly declined only at the lumbar spine in the SG (from 69.0 to 37.9%; *p* = 0.034), whereas no decrease was found in the PG. No severe adverse events occurred.

**Conclusions:**

Postoperative anti-osteoporotic treatment can positively influence regain of BMD mainly in the lumbar spine and should be considered. Without treatment, most patients and especially those with low preoperative markers of bone turnover remained osteoporotic/osteopenic 1 year after surgery.

**Electronic supplementary material:**

The online version of this article (10.1007/s00423-019-01815-9) contains supplementary material, which is available to authorized users.

## Introduction

Primary hyperparathyroidism (pHPT) affects bone metabolism by advancing turnover through increase in both bone formation and resorption, resulting in a net decrease in bone mineral density (BMD) and strength [1, 2]. In the literature, the incidence of osteopenia or osteoporosis in patients with pHPT has been estimated at 39% to 59%, affecting BMD at all sites measured (lumbar spine, femur, and radius) [3–5]. The fracture risk, especially in vertebral spine, is thus increased up to fivefold [6–8] and is associated with low BMD [9]. Parathyroidectomy (PTX) is the only causal treatment for pHPT and is recommended even in patients without severe symptoms, yet reduced BMD [10].

Only three randomized trials have addressed the effect of PTX on BMD vs. observation alone. PTX resulted in a minor but significant increase in BMD of 0.5% to 4% after 1 year [11, 12] and a 3.3% increase only at the lumbar spine after 5 years [13] in patients with mild disease. Improvement of BMD is not found in all patients, as another prospective trial showed no improvement whatsoever following PTX in more than half of postmenopausal women [14]. Thus, the fracture risk is increased by up to 10 or more years following successful surgery [6, 15, 16]. No investigations have so far focused on patients with pHPT and preoperatively diagnosed osteoporosis or osteopenia, who generally are at an advanced risk of fractures, especially not for their postoperative change in BMD and bone turnover.

To our knowledge, no trial has as yet addressed the potentially positive influence of treatment with anti-osteoporotic medication on BMD and bone metabolism after PTX. Basically, these medications can have two physiological effects: they either reduce bone resorption or increase bone formation. While the main effect of bisphosphonate therapy is the reduction of bone resorption, it seems more reasonable to stimulate bone formation to advance the increase in BMD after PTX. Strontium ranelate (SR) is one of the few osteoporosis-specific medications that both stimulate osteoblasts and thereby bone formation and inhibit osteoclasts (reducing bone resorption) [17, 18], leading to a decrease in vertebral and non-vertebral fractures [19, 20]. A meta-analysis of four trials has substantiated the positive effect of 2 g SR daily on the fracture risk in primary osteoporosis [21], [22]. Improved bone microarchitecture [23] may at least partially account for this effect.

Although the use of SR has been restricted (for the potentially elevated risk of thromboembolism and cardiovascular events), it at least continues to serve as an example of an anti-osteoporotic medication for patients with low BMD after successful PTX. The rationale is to stimulate bone formation, re-improve bone strength, increase BMD to the largest extent and as quickly as possible, and thus potentially prevent fractures.

Another new target of pharmacological intervention to increase bone formation is the Wnt signaling pathway, measured by the biochemical parameters sclerostin (SOST) and Dickkopf-1 (DKK-1). Although there is evidence that this pathway is important for the effects of parathyroid hormone (PTH) on bone metabolism [24], the dynamics of SOST and DKK-1 after PTX for PHPT have not yet been investigated. This pathway is of special interest, as the novel therapeutic agent romosozumab, an antibody against SOST, has shown potent anti-osteoporotic effects [25].

Thus, the aim of this study was to focus on osteopenic and osteoporotic patients after PTX for pHPT in an attempt to investigate the effect of an anti-osteoporotic medication with an anabolic effect on the change of BMD and on classical biochemical markers of bone metabolism. Additionally, the parameters SOST and DKK-1 were analyzed as potential future targets of intervention for stimulating bone formation.

## Materials and methods

### Patients

All postmenopausal women and men with biochemically proven pHPT and osteopenia (T-score ≤ 1 and ≥ 2.5) or osteoporosis (T-score ≤ 2.5) according to WHO criteria [26] consulting the Department of Surgery, Medical University of Vienna, were asked to participate in this study prior to PTX. Following the recently published guidelines for the diagnosis and definitive management of pHPT, all patients fulfilled the criteria for surgical intervention [10, 27–29].

The exclusion criteria before surgery included anamnestic pulmonary embolism or deep venous thrombosis, blood coagulation disorder or coagulopathy, phenylketonuria, renal impairment (creatinine clearance < 30 mL/h), severe hepatic disorder, severe systemic disorder, thyroid dysfunction, and immobilization. Additionally, the intake of drugs with potential effects on BMD, such as glucocorticoids, lithium, estrogen replacement therapy, selective estrogen receptor modulators, bisphosphonates (oral: last 3 months, parenteral: past year), and denosumab (past year), was not allowed. The patients were excluded after surgery in the case of malignant disease (thyroid or parathyroid cancer, except for microcarcinoma of the thyroid gland), persistent or recurrent pHPT (postoperative hypercalcemia), four-gland hyperplasia, multiple endocrine neoplasia, hereditary pHPT, or familial hypocalcuric hypercalcemia (calcium/creatinine ratio < 0.01). In accordance with European Medicines Agency regulations, additional exclusion criteria (ischemic cardiac disease, peripheral arterial obstructive disease, cerebrovascular disease, and uncontrolled arterial hypertonia) were introduced in April 2013. Since then, electrocardiograms have been included in prestudy screening and were also performed after the 12-month study period.

The study procedures of this double-blind, placebo-controlled, randomized trial were approved by the Ethics Committee of the Medical University of Vienna (EKNr: 2142008), the Austrian Agency for Health and Food Safety (Österreichische Agentur für Gesundheit und Ernährungssicherheit), ClinicalTrials.gov (identifier: NCT01222026), and European Union Drug Regulating Authorities Clinical Trials. All study participants gave written informed consent in accordance with the Declaration of Helsinki.

### Study design and treatment protocol

PTX was performed according to the current indications for surgery [10, 27]. Within 1 week after surgery, all enrolled patients received daily supplements of 1000 mg of calcium and 800 IU of vitamin D. Four weeks after surgery, the patients were randomly assigned to be given placebo (placebo group; PG) or SR 2 g daily (strontium group; SG) for 1 year (sequentially numbered containers). The primary outcome variable was BMD at the lumbar spine. The secondary outcome parameters were BMD at other sites and biochemical parameters.

### BMD

The BMD measurements at the lumbar spine, left femoral neck, and non-dominant radius (one-third distal [1/3 radius], mid-distal [MID radius], and ultradistal [UD radius]) were performed using a dual-energy X-ray absorption (DXA) device (HOLOGIC 4500; Hologic Inc., Waltham, MA, USA; coefficient of variation: 2%). All measurements were conducted using the standard procedures recommended by the manufacturer. The BMD values are expressed as g/cm^2^ and T-scores.

### Biochemical parameters

Overnight fasting venous blood samples were taken before surgery, 4 weeks, and 3, 6, and 12 months after surgery. Routine parameters (PTH, ionized calcium [Ca^++^], phosphate, 25-hydroxyvitamin D [25(OH)D], 1,25-dihydroxyvitamin D [1,25(OH)D]) were determined using standard methods. Additionally, bone turnover markers were studied: bone-specific alkaline phosphatase (BAP; Liaison Analyzer, DiaSorin Inc., Stillwater, MN, USA; detection limit: 0.1 μg/L; intra-assay coefficient of variation: 3.3–4.3%, inter-assay coefficient of variation: 6.1–8.1%), osteocalcin (OC; Cobas 8000 Analyzer, Roche Diagnostics, Rotkreuz, Switzerland; detection limit: 0.01 ng/mL; intra-assay coefficient of variation: 0.9–1.3%, inter-assay coefficient of variation: 1.2–2.3%), and CrossLaps (CTX; Cobas 8000 Roche Analyzer, Roche Diagnostics, Rotkreuz, Switzerland; detection limit: 0.5 ng/mL; intra-assay coefficient of variation: 1.2–4.7%, inter-assay coefficient of variation: 1.5–5.7%). Serum was frozen at − 70 °C until analysis for osteoprotegerin (OPG, BI-20403; colorimetric sandwich immunoassays, Biomedica, Vienna, Austria; detection limit: 0.07 pmol/L; intra-assay coefficient of variation: ≤ 3%, inter-assay coefficient of variation: ≤ 5%, according to the manufacturer’s data), receptor activator of nuclear factor-kappa B ligand (RANKL, BI-20452; colorimetric sandwich immunoassays, Biomedica, Vienna, Austria; detection limit: 0.02 pmol/L; intra-assay coefficient of variation: ≤ 9%; inter-assay coefficient of variation: ≤ 6%, according to the manufacturer’s data), SOST (BI-20492; colorimetric sandwich immunoassays, Biomedica, Vienna, Austria; detection limit: 2.6 pmol/L; intra-assay coefficient of variation: ≤ 5%; inter-assay coefficient of variation: ≤ 6%, according to the manufacturer’s data), and DKK-1 (BI-20412; colorimetric sandwich immunoassays, Biomedica, Vienna, Austria; detection limit: 0.38 pmol/L; intra-assay coefficient of variation: ≤ 8.0%, inter-assay coefficient of variation: ≤ 12.0%, according to the manufacturer’s data).

### Statistical analysis

Normal distribution was assessed with the Shapiro-Wilk test and visual inspection of histograms. After descriptive analysis, parametric tests (*t* test for baseline characteristics and BMD) and non-parametric tests (Wilcoxon’s rank-sum test for biochemical parameters) were used for group comparison. In order to compare the two time points, longitudinal changes within each group were evaluated with the paired *t* test (BMD) or the Wilcoxon’s signed rank test (biochemical parameters). Fisher’s exact test was used to test for differences in binominal proportions. As the sample size was inadequate for the Chi-square test of homogeneity, Fisher’s exact test was also applied to test for differences in multinomial distributions. Spearman’s rank order correlation was run to assess the relationship between the increase in BMD and preoperative biochemical parameters.

Data are given as mean (± standard deviation [SD]) or median (25th quartile; 75th quartile). *P* values < 0.05 were considered significant.

### Sample size

The sample size was calculated in cooperation with the Institute of Medical Statistics, Medical University of Vienna, to provide a statistical power of 80% accepting an alpha error of 5%. Background data were taken from the literature. The 1-year treatment effect of SR was estimated as 3% BMD change in the lumbar spine. The SD in the literature was ± 3.7% [19, 20]. Thirty patients were to be recruited in each group (including an estimated 20% loss of follow-up).

## Results

### Baseline parameters

**Fig. 1:**
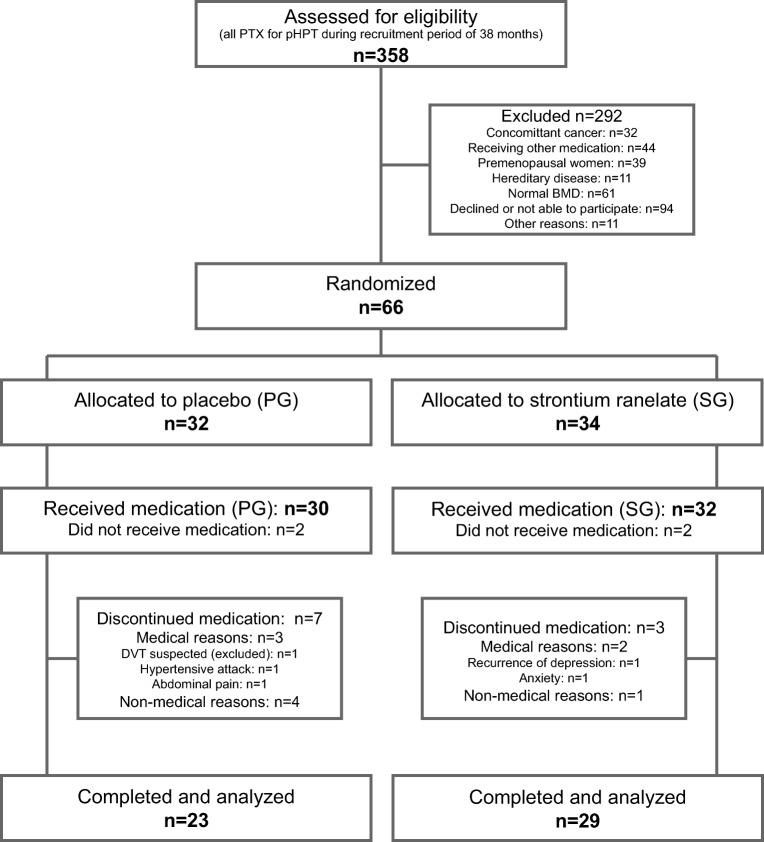
Flow diagram of screening, randomization, and completion

Of the 358 patients who underwent PTX due to biochemically proven pHPT at our institution during the study period, 291 were ineligible or not interested in participating (Fig. 1). Four of the 66 randomly assigned subjects (SG: 34; PG: 32) were excluded before they received medication because of hypercalcemia and intolerance of calcium supplementation. The study medication was finally administered to 62 patients. Patient withdrawals were due to medical reasons (SG: 2; PG: 3), protocol violations (PG: 2), and nonmedical reasons (SG: 1; PG: 2). Thus, 29 patients in the SG and 23 in the PG were included in the statistical analysis (per protocol).

Of these 52 patients, 8 had typical and 44 only minor or no symptoms of pHPT. Open minimally invasive PTX (OMIP) was performed in 20 patients, unilateral exploration for PTX in 8 patients, and bilateral exploration for PTX in 24 patients. In most cases (*n* = 50), histological analysis revealed an adenoma of the parathyroid gland; only two patients had hyperplasia.

The baseline characteristics were similar and did not differ significantly between the groups. There were no surgical complications (Table 1).

**Table 1:** Baseline characteristics; mean (± SD) or median (25th percentile; 75th percentile)

	Placebo group (*n* = 23)	Strontium group (*n* = 29)	*p*
Gender f/m	16/7	16/13	
Type of operation			
OMIP	10	11	
Unilateral exploration	2	6	
Bilateral exploration	11	12	
Surgical complications			
Bleeding	0	0	
Recurrent nerve palsy	0	0	
Age (years)	63 (± 10)	63 (± 12)	0.949
Height (cm)	168 (± 8)	170 (± 10)	0.481
Weight (kg)	76 (± 15)	80 (± 16)	0.388
BMI	27.1 (± 4.8)	27.8 (± 4.4)	0.561
Baseline BMD lumbar spine (g/cm^2^)	0.864 (± 0.134)	0.917 (± 0.185)	0.260
Baseline T-score lumbar spine	− 1.8 (± 1.2)	− 1.4 (± 1.5)	0.300
Baseline BMD femoral neck (g/cm^2^)	0.680 (± 0.123)	0.686 (± 0.114)	0.867
Baseline T-score femoral neck	− 1.6 (± 1.1)	− 1.7 (± 0.9)	0.953
Baseline BMD 1/3 radius (g/cm^2^)	0.585 (0.085)	0.605 (± 0.088)	0.410
Baseline T-score 1/3 radius	− 2.4 (± 1.2)	− 2.4 (± 1.0)	0.821
Baseline BMD MID radius (g/cm^2^)	0.512 (± 0.077)	0.520 (± 0.080)	0.718
Baseline T-score MID radius	− 2.3 (± 1.2)	− 2.4 (± 1.0)	0.815
Baseline BMD UD radius (g/cm^2^)	0.396 (± 0.091)	0.390 (± 0.077)	0.805
Baseline T-score UD radius	− 1.6 (± 1.1)	− 1.7 (± 0.9)	0.601
PTH (pg/mL)	123.9 (90.8; 162.2)	141.3 (97.4; 162.9)	0.747
Ca^++^ (mmol/L)	1.39 (1.36; 1.43)	1.41 (1.38; 1.50)	0.264
Phosphate (mmol/L)	0.70 (0.57; 0.83)	0.64 (0.57; 0.74)	0.342
25(OH)D (nmol/L)	38.9 (26.8; 47.4)	39.8 (29.8; 52.1)	0.719
1,25(OH)D (nmol/L)	62.0 (49.0; 77.0)	64.0 (54.0; 96.0)	0.612
BAP (ng/mL)	20.4 (14.6; 28.3)	15.9 (10.8; 27.7)	0.339
OC (ng/mL)	41.8 (22.8; 59.6)	33.2 (25.2; 43.3)	0.315
CTX (ng/mL)	0.84 (0.42; 1.01)	0.63 (0.52; 0.79)	0.325
P1NP (ng/mL)	61.5 (47.0; 89.0)	52.0 (43.0; 74.5)	0.482
24 h CrCl (mL/min)	107.7 (96.4; 132.8)	121.6 (89.4; 161.5)	0.768
Patients with 24 h CrCl < 60 mL/min (*n*)	0	2^a^	
OPG (pmol/L)	4.46 (3.64; 5.64)	4.65 (3.63; 5.47)	0.869
RANKL (pmol/L)	0.87 (0.51; 1.42)	0.74 (0.52; 0.99)	1
SOST (pmol/L)	19.63 (15.55; 29.31)	20.84 (14.85; 28.49)	0.934
DKK-1 (pmol/L)	25.10 (18.99; 35.69)	32.32 (23.71; 41.04)	0.129

^a^Both patients had a 24 h CrCl of > 60 mL/min 1 year after surgery

*OMIP* open minimally invasive parathyroidectomy, *BMI* body mass index, *BMD* bone mineral density, *1/3 radius* one-third distal radius, *MID radius* Mid-distal radius, *UD radius* ultradistal radius, *PTH* parathyroid hormone, *Ca*^*++*^ ionized calcium, *25(OH)D* 25-hydroxyvitamin D, *1,25(OH)D* 1,25-dihydroxyvitamin D, *BAP* bone-specific alkaline phosphatase, *OC* osteocalcin, *CTX* CrossLaps, *P1NP* procollagen type 1 N-terminal propeptide, *24 h CrCl* measured 24-h creatinine clearance, *OPG* osteoprotegerin, *RANKL* receptor activator of nuclear factor-kappa B ligand, *SOST* sclerostin, *DKK-1* Dickkopf-1

### Change in BMD

At the lumbar spine, absolute BMD after 1 year was higher in the SG than in the PG (1.007 ± 0.197 g/cm^2^ vs. 0.897 ± 0.137 g/cm^2^; *p* = 0.024). Additionally, the percentage change of BMD after 1 year was more than twofold higher in the SG than in the PG (9.94 vs. 3.94%; *p* < 0.001). Also, the absolute BMD change was significantly higher in the SG (0.09 ± 0.06 g/cm^2^ vs. 0.03 ± 0.04 g/cm^2^; *p* < 0.001). At all other sites, both the percentage and absolute changes in BMD were higher in the SG but did not reach the level of significance (Table 2).

**Table 2:** Relative changes (Δ) in BMD after 1 year for both (female + male), female and male patients: % (± SD)

	Placebo group	Strontium group	*p*
Δ BMD lumbar spine (%)			
Both	3.94 (± 4.49)	9.94 (± 6.33)	< 0.001
Female	4.98 (±4.28)	10.71 (± 6.18)	0.005
Male	1.73 (± 4.42)	8.99 (± 6.63)	0.009
Δ BMD femoral neck (%)			
Both	4.84 (± 4.55)	5.87 (± 5.86)	0.504
Female	5.62 (± 4.83)	6.21 (± 6.28)	0.772
Male	3.27 (± 3.75)	5.42 (± 5.49)	0.326
Δ BMD 1/3 radius (%)			
Both	0.00 (± 3.36)	0.42 (± 4.06)	0.690
Female	0.75 (± 3.00)	0.40 (± 4.80)	0.810
Male	− 1.61 (± 3.76)	0.45 (± 3.11)	0.241
Δ BMD MID radius (%)			
Both	0.41 (± 2.86)	1.64 (± 3.23)	0.166
Female	0.97 (± 2.29)	2.09 (± 3.71)	0.316
Male	− 0.78 (± 3.75)	1.08 (± 2.57)	0.271
Δ BMD UD radius (%)			
Both	1.84 (± 5.76)	3.02 (± 5.86)	0.474
Female	2.90 (± 5.37)	4.37 (± 6.14)	0.482
Male	− 0.43 (± 6.33)	1.37 (± 5.26)	0.535

*SD* standard deviation, *BMD* bone mineral density, *1/3 radius* one-third distal radius, *MID radius* mid-distal radius, *UD radius* ultradistal radius

**Fig. 2:**
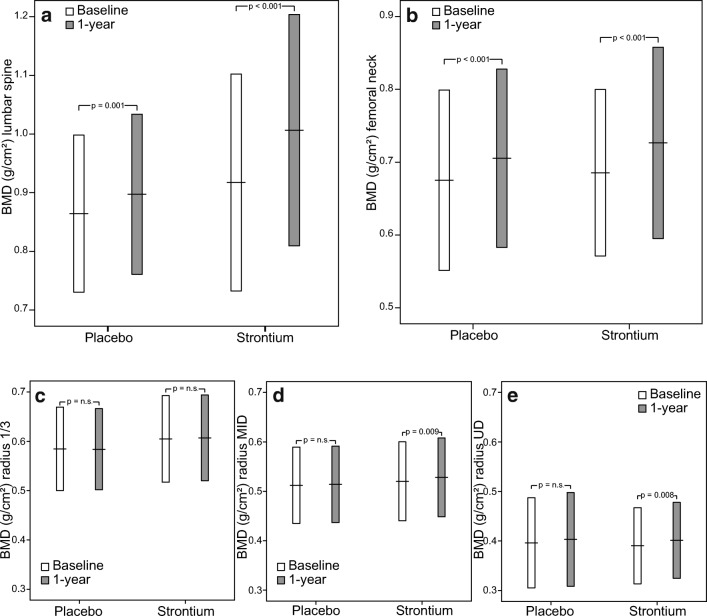
Bone mineral density (BMD; mean ± 1SD) at baseline and after 1 year in lumbar spine (**a**), femoral neck (**b**), one-third distal radius (1/3 radius, **c**), mid-distal radius (MID radius, **d**), and ultradistal radius (UD radius, **e**)

Comparing baseline and 1-year controls in both the SG and the PG, there was a significant increase in BMD at the lumbar spine (both *p* < 0.001) and the femoral neck (*p* < 0.001 and *p* = 0.001). Radius BMD (except 1/3 radius) changed significantly in the SG only (*p* = 0.008 and 0.009, respectively) (Fig. 2, Supplementary Data 1). Both the percentages and absolute changes did not differ significantly between female and male patients, neither in the SG nor in the PG.

When summing up osteoporosis and osteopenia, the percentage of lumbar-spine disorders before treatment was 68.9% in the SG and 77.3% in the PG. A statistically significant decline to 37.9% (*p* = 0.034) was seen in the SG, whereas no relevant change was found in the PG (72.7%; *p* = 1.000). Although statistically not significant anymore, there was a marked increase in patients with normal BMD values at the lumbar spine in the SG (from 31.0% at baseline to 62.1% after 1 year, *p* = 0.061), whereas this increase was not found in the PG (from 22.7% at baseline to 27.6% after 1 year, *p* = 0.734; Supplementary Data 2).

Initial osteoporosis and osteopenia at the femoral neck were identified in 89.3% and 68.2% of the patients in the SG and the PG, respectively. After the 1-year follow-up period, there was a trend towards a decrease in osteoporotic/osteopenic patients in the SG with a 17.9% reduction to 71.4% (*p* = 0.177). This reduction was not found in the PG (66.7% after 1 year; *p* = 1.000).

At the UD radius, the changes from 79.3 to 65.5% in the SG and from 81.8 to 73.9% in the PG did not prove to be statistically different (*p* = 0.379 and 0.722, respectively). At the MID radius and 1/3 radius, the percentages of osteoporosis and osteopenia remained nearly unchanged in both groups at more than 80% (*p* = 1.000 for both; *p* = 1.000 and *p* = 0.491, respectively).

### Change in biochemical parameters

The PTH and Ca^++^ levels decreased and phosphate increased as indices of successful pHPT treatment. Comparing the groups after 1 year, no statistically significant differences were seen except for the Ca^++^ levels: the decrease in Ca^++^ levels was significantly higher in the SG compared to the PG (− 0.22 vs. − 0.12 mmol/L; *p* = 0.004) (Table 3).

**Table 3:** Biochemical parameters: intra-group changes (baseline to 1 year) and comparison between groups after 1 year

	Placebo group			Strontium group			Between-group after 1 year
	Baseline	1 year	*p*	Baseline	1 year	*p*	*p*
PTH (pg/mL)	123.9 (90.8; 162.2)	36.2 (15.9; 51.1)	< 0.001	141.3 (97.4; 162.9)	29.5 (± 23.5; 41.0)	< 0.001	0.362
Ca++ (mmol/L)	1.39 (1.36; 1.43)	1.25 (1.21; 1.28)	< 0.001	1.41 (1.38; 1.50)	1.22 (1.18; 1.24)	< 0.001	0.004
Phosphate (mmol/L)	0.70 (0.57; 0.83)	1.03 (0.90; 1.17)	< 0.001	0.64 (0.57; 0.74)	1.04 (0.95; 1.25)	< 0.001	0.265
25(OH)D (nmol/L)	38.9 (26.8; 47.4)	76.1 (61.6; 84.3)	< 0.001	39.8 (29.8; 52.1)	67.9 (54.7; 83.4)	< 0.001	0.352
1,25(OH)D (nmol/L)	62.0 (49.0; 77.0)	46.0 (40.0; 65.0)	0.017	57.0 (41.0; 73.0)	57.0 (41.0; 73.0)	0.011	0.525
BAP (ng/mL)	20.4 (14.6; 28.3)	11.4 (8.1; 12.4)	0.01	15.9 (10.8; 27.7)	9.3 (8.1; 12.1)	0.001	0.410
OC (ng/mL)	41.8 (22.8; 59.6)	14.9 (12.7; 20.8)	< 0.001	33.2 (25.2; 43.3)	14.3 (11.6; 17.8)	< 0.001	0.519
CTX (ng/mL)	0.84 (0.42; 1.01)	0.19 (0.15; 0.28)	< 0.001	0.63 (0.52; 0.79)	0.16 (0.14; 0.24)	< 0.001	0.366
P1NP (ng/mL)	61.5 (47.0; 89.0)	24.0 (20.0; 31.0)	< 0.001	52.0 (43.0; 74.5)	24.0 (22.0; 31.0)	< 0.001	0.890
24 h CrCl (mL/min)	107.7 (96.4; 132.8)	91.4 (78.9; 129.0)	0.05	121.6 (89.4; 161.5)	112.6 (90.6; 136.2)	0.194	0.113
OPG (pmol/L)	4.46 (3.64; 5.64)	4.97 (3.57; 7.48)	0.087	4.65 (3.63; 5.47)	5.05 (3.38; 6.27)	0.976	0.525
RANKL (pmol/L)	0.87 (0.51; 1.42)	0.58 (0.37; 1.35)	0.465	0.74 (0.52; 0.99)	0.65 (0.42; 0.94)	0.068	1
SOST (pmol/L)	19.63 (15.55; 29.31)	20.33 (16.16; 41.01)	0.043	20.84 (14.85; 28.49)	21.28 (18.42; 29.01)	0.121	0.920
DKK-1 (pmol/L)	25.10 (18.99; 35.69)	34.07 (20.06; 39.39)	0.008	32.32 (23.71; 41.04)	34.40 (29.39; 45.43)	0.021	0.221

*PTH* parathyroid hormone, *Ca*^*++*^ ionized calcium, *25(OH)D* 25-hydroxyvitamin D, *1,25(OH)D* 1,25-dihydroxyvitamin D, *BAP* bone-specific alkaline phosphatase, *OC* osteocalcin, *CTX* CrossLaps, *P1NP* procollagen type 1 N-terminal propeptide, *24 h CrCl* measured 24-h creatinine clearance, *OPG* osteoprotegerin, *RANKL* receptor activator of nuclear factor-kappa ligand, *SOST* sclerostin, *DKK-1* Dickkopf-1

The markers of bone formation and markers of bone resorption decreased significantly in the SG and the PG (Table 3). Both parameters of the Wnt signaling pathway showed an increase: DKK-1 increased significantly in both groups and SOST showed a small increase in both groups that was only significant in the PG (*p* = 0.043 vs. *p* = 0.121 in the SG; Table 3).

### Effects of preoperative PTH on biochemical markers of bone metabolism

At baseline, the PTH levels were positively correlated with Ca^++^ (*r* = 0.496; *p* < 0.001), OC (*r* = 0.482; *p* < 0.001), CTX (*r* = 0.420; *p* < 0.005), and procollagen type 1 N-terminal propeptide (P1NP; *r* = 0.332; *p* < 0.05), but not with BAP, OPG, RANKL, SOST, or DKK-1.

### Correlation of BMD increase with preoperative parameters

In the PG, there was a strong and positive correlation between the preoperative levels of OC, P1NP, and CTX and the increase in lumbar-spine BMD (PG: *r* = 0.634, *r* = 0.750, *r* = 0.634, respectively). This correlation was weaker in the SG (SG: *r* = 0.413, *r* = 0.329, *r* = 0.329, respectively) (Table 4). At the femoral neck, a relevant correlation between BAP and increase in BMD (*r* = 0.510) was only identified in the PG.

**Table 4:** Correlation between absolute change in lumbar spine BMD and preoperative levels of markers of bone metabolism; *R*: Spearman correlation index

	Placebo group		Strontium group	
	Spearman index (*R*)	*p*	Spearman index (*R*)	*p*
Age	0.085	0.706	0.139	0.473
PTH	0.292	0.187	0.232	0.226
Ca^++^	0.292	0.187	0.256	0.180
OC	*0.634*	*0.002*	0.352	0.061
P1NP	*0.750*	< *0.001*	*0.413*	*0.029*
CTX	*0.634*	*0.002*	0.274	0.159
BAP	0.229	0.413	*0.488*	*0.029*
OPG	− 0.116	0.627	− 0.162	0.450
RANKL	0.600	0.400	− 0.800	0.200
SOST	0.098	0.682	0.079	0.713
DKK-1	− 0.030	0.904	− 0.206	0.335

*BMD* bone mineral density, *PTH* parathyroid hormone, *Ca*^*++*^ ionized calcium, *OC* osteocalcin, *P1NP* procollagen type 1 N-terminal propeptide, *CTX* CrossLaps, *BAP* bone-specific alkaline phosphatase, *OPG* osteoprotegerin, *RANKL* receptor activator of nuclear factor-kappa ligand, *SOST* sclerostin, *DKK-1* Dickkopf-1

No significant correlation was seen between the preoperative levels of PTH, OPG, RANKL, SOST, or DKK-1 and gain in BMD at any site measured.

### Adverse events

The intake of study medication did not induce any adverse events. In particular, no patient in either group developed cardiovascular disorders or thromboembolic complications during the study. No cases of vertebral or non-vertebral fractures occurred.

## Discussion

This is the first prospective, randomized, double-blind trial to investigate the effect of anti-osteoporotic treatment on the postoperative course of BMD and bone metabolism after successful PTX, focusing only on patients with preoperatively advanced bone involvement (diagnosed osteoporosis or osteopenia). The rationale of this intervention was to enhance the speed and extent of bone density normalization in the early postoperative course. With intervention, the percentage of BMD increase at the lumbar spine was more than twofold higher in the treatment group than in the placebo group and the percentage of patients with osteopenia or osteoporosis declined from 69.0 to 37.9%. The additional effect of 6% lumbar-spine BMD change (PG: 3.94%; SG: 9.94%) shown in this study was equal to the 1-year baseline change after SR intake found in patients with primary osteoporosis but without pHPT (postmenopausal women and men). This treatment effect has been seen to reduce both vertebral and non-vertebral fractures [19, 20, 30]. As the fracture risk is prolonged up to 10 or more years following successful PTX in patients with pHPT [6, 15, 16], this effect seems preferable. The data presented here thus demonstrate that an anti-osteoporotic medication may additionally and positively influence bone remineralization, while producing similar effects as in patients without preceding PTX for pHPT. The effect on lumbar-spine BMD seems to be of even greater importance, as this site appears to be more strongly affected by pHPT [31, 32].

Although statistically not significant, a tendency towards a more pronounced gain in BMD at all sites was observed in females compared to males in either group (see Table 2). Especially for postmenopausal women who per se are at an elevated risk of fractures, any positive effect on bone stability seems important, since this trial showed that surgical cure of pHPT without additional treatment failed to reduce the proportion of patients with osteoporosis or osteopenia 1 year after PTX at any site in spite of adequate vitamin D and calcium supplementation. In a prospective and another retrospective trial, a further decrease in BMD was even documented in 15% to 31% [14, 33].

The information on biochemical markers presented here offers a broad insight into postoperative changes following PTX. Basically, the levels of Ca^++^ at diagnosis were only slightly elevated (1.39 and 1.41 mmol/L in the SG and PG, respectively; normal range 1.16–1.32 mmol/L) as consequence to earlier diagnoses in Western countries due to screening programs with more frequently asymptomatic patients [31]. Nevertheless, alterations in markers of bone metabolism were documented in all patients demonstrating relevant bone involvement, albeit with a high level of variability (thus suggesting individual differences in pHPT-induced bone affection). The serum levels of CTX, a bone resorption marker, as well as those of the bone formation markers OC and P1NP significantly declined in all subjects at follow-up compared to baseline (Table 3). Additionally, the two antagonists of the Wnt signaling pathway (“antagonists” of bone formation)—SOST and DKK-1—that have not yet been evaluated following PTX, slightly increased in both treatment groups over the 1-year follow-up. Both the increase in SOST and DKK-1 and the reduction of “classical” markers of bone metabolism suggest that PTX reduces bone resorption as well as bone formation. These findings are in line with our previous study, also showing reduced bone turnover after PTX surgery [34, 35]. Therefore, it seems unreasonable to postoperatively apply a mainly antiresorptive treatment such as bisphosphonates that have even shown to bear potentially negative effects in pHPT-related bone involvement [36]. Although SR was limited in its use in 2017 (due to an advanced risk of thromboembolism and cardiovascular events), it seems reasonable to consider other anti-osteoporotic medications stimulating bone formation to improve BMD after PTX. Alternatively, the Wnt signaling pathway may be an interesting starting point for further investigation, as both SOST and DKK-1 levels were seen to increase postoperatively in this trial. Romosozumab, an antibody against SOST, is a promising new therapeutic agent against osteoporosis, improving bone formation, and microarchitecture [25]. It was recently approved by the FDA and might be another potential pharmacological option. This assumption is in line with findings that expression of SOST in bone is enhanced after PTX for secondary (renal) HPT [37].

In addition to the effect of PTH in pHPT, low BMD may also be caused by such other factors as primary osteoporosis, especially in postmenopausal women. We therefore attempted to establish markers of bone metabolism that may potentially reflect bone involvement by pHPT. OC and CTX were strongly correlated (as strongly as Ca^++^) with preoperative PTH levels, and both markers, yet not PTH, showed a correlation with postoperative increase in BMD at the lumbar spine. These findings are in accordance with the study authored by Hansen et al., showing a correlation between increase in volumetric BMD and CTX (no data for OC) [38], but contradict the findings of Rolighed et al. who detected a positive association between preoperative PTH levels and postoperative BMD increase [39]. Preoperative biochemical markers may help to identify patients in whom a pronounced positive effect of successful PTX on postoperative BMD may be expected. They may also assist in differentiating such patients from those in whom primary osteoporosis and other factors may additionally strongly affect bone quality and who are less likely to improve after cure of pHPT, thus requiring immediate additional therapy.

Essentially, our investigation had several strengths: it was the first to investigate the effect of an anti-osteoporotic treatment immediately after successful PTX. Apart from its prospective, randomized, and double-blind design, the study population was very homogeneous, including only osteopenic and osteoporotic patients. Moreover, all patients were supplied with adequate standardized vitamin D and calcium doses, as shown to be important after PTX for pHPT [40].

A limitation of this trial is that SR was restricted in its use at the end of patient recruitment. Some of the measured effect of SR is likely caused by the overestimation of BMD in DXA scans of patients treated with SR (due to the higher atomic number of strontium compared to calcium [41]). However, even if 25% of the additional effect (approx. 6%) in the SG was caused by overestimation of BMD, the increase would still be 3.5% higher than in the PG (3.94% vs. SG: 7.47%; *p* = 0.01).

## Conclusion

In conclusion, this randomized, double-blind, placebo-controlled trial indicated that the treatment of osteopenic or osteoporotic patients following PTX increases BMD mainly at the lumbar spine. The rationale of this trial was emphasized by the fact that without treatment, nearly all patients remained osteoporotic or at least osteopenic even with an adequate supply of vitamin D and calcium. In patients with high levels of OC and CTX, an increase in BMD is more likely than in those with low markers of bone metabolism. Thus, at least patients with osteoporosis/osteopenia with a lower probability of improvement after PTX should either be monitored closely for improvement in bone density or even receive bone-specific treatment immediately after PTX to reduce the risk of potential complications. As strontium ranelate was restricted in its use, other medications with positive effects on bone formation should be evaluated and the Wnt pathway may be another target for therapy.

## Electronic supplementary material

ESM 1 (DOCX 19 kb).

## Abbreviations

SG: Strontium group
PG: Placebo group
pHPT: Primary hyperparathyroidism
BMD: Bone mineral density
PTX: Parathyroidectomy
PTH: Parathyroid hormone
Ca^++^: Ionized calcium
25(OH)D: 25-hydroxyvitamin D
1,25(OH)D: 1,25-dihydroxyvitamin D
BAP: Bone-specific alkaline phosphatase
CTX: CrossLaps
P1NP: Procollagen type 1 N-terminal propeptide
24 h CrCl: Measured 24-h creatinine clearance
OPG: Osteoprotegerin
RANKL: Receptor activator of nuclear factor-kappa B ligand
DKK-1: Dickkopf-1
1/3 radius: One-third distal radius
MID radius: Mid-distal radius
UD radius: Ultradistal radius
DXA: Dual-energy X-ray absorption
OC: Osteocalcin
SD: Standard deviation
SOST: Sclerostin
SR: Strontium ranelate
OMIP: Open minimally invasive parathyroidectomy
