# Supplementary material for: Effectiveness of anti-osteoporotic treatment after successful parathyroidectomy for primary hyperparathyroidism: a randomized, double-blind, placebo-controlled trial
Source: Langenbecks Arch Surg. 2019 Aug 26;404(6):681–91. doi: 10.1007/s00423-019-01815-9 (PMC6906217; doi:10.1007/s00423-019-01815-9)
Supplement: Supplementary file 1 — (DOCX 19 kb). [file 423_2019_1815_MOESM1_ESM.docx]

**Supplementary Data:**

Supplementary Data 1: Absolute BMD (mean ± SD) at baseline and after 1 year

|  | **Placebo group** | | | **Strontium group** | | |
| --- | --- | --- | --- | --- | --- | --- |
|  | **Baseline** | **1 year** | **p** | **Baseline** | **1 year** | **p** |
| **BMD lumbar spine (g/cm²)** | 0.864 (±0.134) | 0.897  (±0.137) | 0.001 | 0.917 (±0.185) | 1.007  (±0.197) | <0.001 |
| **T-score lumbar spine** | -1.8  (±1.2) | -1.4  (±1.2) | <0.001 | -1.4  (±1.5) | -0.5  (±1.7) | <0.001 |
| **BMD femoral neck (g/cm²)** | 0.680 (±0.123) | 0.705  (± 0.122) | <0.001 | 0.686 (±0.114) | 0.726  (±0.131) | <0.001 |
| **T-score femoral neck** | -1.6  (±1.1) | -1.4  (±1.1) | <0.001 | -1.7  (±0.9) | -1.3  (±1.0) | <0.001 |
| **BMD 1/3 radius (g/cm²)** | 0.585 (±0.085) | 0.584  (±0.082) | 0.873 | 0.605 (±0.088) | 0.509  (±0.080) | 0.647 |
| **T-score 1/3 radius** | -2.4  (±1.2) | -2.4  (± 1.2) | 0.847 | -2.4  (±1.0) | -2.4  (±0.8) | 0.946 |
| **BMD MID radius (g/cm²)** | 0.512 (±0.077) | 0.514  (±0.077) | 0.556 | 0.520 (±0.080) | 0.528  (±0.080) | 0.009 |
| **T-score MID radius** | -2.3  (±1.2) | -2.3  (±1.2) | 0.141 | -2.4  (±1.0) | -2.2  (±1.0) | 0.002 |
| **BMD UD radius (g/cm²)** | 0.396 (±0.091) | 0.403  (± .095) | 0.185 | 0.390 (±0.077) | 0.401  (±0.077) | 0.008 |
| **T-score UD radius** | -1.6  (±1.1) | -1.4  (±1.1) | 0.025 | -1.7  (±0.9) | -1.5  (±1.0) | 0.001 |

BMD: Bone mineral density; SD: Standard deviation; 1/3 radius: one-third distal radius; MID radius: mid-distal radius; UD radius: ultradistal radius

Supplementary Data 2: Distribution of patients with normal BMD, osteopenia or osteoporosis at all sites comparing baseline to status after 1 year

| **Site** | **Group** | **Baseline** | | | **1 year** | | | **p** |
| --- | --- | --- | --- | --- | --- | --- | --- | --- |
|  |  | **Normal**  **n (%)** | **Osteo-penia**  **n (%)** | **Osteo-porosis**  **n (%)** | **Normal**  **n (%)** | **Osteo-penia**  **n (%)** | **Osteo-porosis**  **n (%)** |  |
| **Lumbar spine*** | Placebo | 5 | 9 | 8 | 6 | 11 | 5 | 0.734 |
|  |  | (22.7%) | (40.9%) | (36.4%) | (27.3%) | (50.0%) | (22.7%) |  |
|  | Strontium | 9 | 13 | 7 | 18 | 8 | 3 | 0.061 |
|  |  | (31.0%) | (44.8%) | (24.1%) | (62.1%) | (27.6%) | (10.3%) |  |
| **Femoral neck** | Placebo | 7 | 9 | 6 | 7 | 10 | 4 | 0.858 |
|  |  | (31.8%) | (40.9%) | (27.4%) | (33.3%) | (47.6%) | (19.0%) |  |
|  | Strontium | 3 | 21 | 4 | 8 | 18 | 2 | 0.243 |
|  |  | (10.7%) | (75.0%) | (14.3%) | (28.6%) | (64.3%) | (7.1%) |  |
| **1/3 radius** | Placebo | 3 | 9 | 10 | 3 | 8 | 12 | 0.917 |
|  |  | (13.6%) | (40.9%) | (45.5%) | (13.0%) | (34.8%) | (52.2%) |  |
|  | Strontium | 2 | 15 | 12 | 0 | 14 | 15 | 0.352 |
|  |  | (6.9%) | (51.7%) | (41.4%) | (0%) | (48.3%) | (51.7%) |  |
| **MID radius** | Placebo | 3 | 4 | 7 | 11 | 5 | 7 | 1.000 |
|  |  | (13.0%) | (18.2%) | (31.8%) | (50.0%) | (21.7%) | (30.4%) |  |
|  | Strontium | 5 | 2 | 15 | 12 | 2 | 17 | 0.915 |
|  |  | (17.2%) | (6.9%) | (51.7%) | (41.4%) | (6.9%) | (58.6%) |  |
| **UD radius** | Placebo | 4 | 14 | 4 | 6 | 14 | 3 | 0.834 |
|  |  | (18.2%) | (63.6%) | (18.2%) | (26.1%) | (60.9%) | (13.0%) |  |
|  | Strontium | 6 | 17 | 6 | 10 | 14 | 5 | 0.532 |
|  |  | (20.7%) | (58.6%) | (20.7%) | (34.5%) | (48.3%) | (17.2%) |  |

* Distribution equal at baseline between placebo and strontium group (p=0.327)

1/3 radius: one-third distal radius; MID radius: mid-distal radius; UD radius: ultradistal radius
